# Hospital pharmacists’ knowledge of and attitudes towards the implementation of the National Essential Medicines System: a questionnaire survey in western China

**DOI:** 10.1186/s12913-016-1537-9

**Published:** 2016-07-20

**Authors:** Qian Shen, Caijun Yang, Jie Chang, Lina Wu, Wenwen Zhu, Bing Lv, Dan Ye, Shimin Yang, Yu Fang

**Affiliations:** Department of Pharmacy Administration and Clinical Pharmacy, School of Pharmacy, Xi’an Jiaotong University, No. 76 West Yanta Road, Xi’an, Shaanxi 710061 China; Center for Drug Safety and Policy Research, Xi’an Jiaotong University, Xi’an, Shaanxi 710061 China

**Keywords:** National Essential Medicines System (NEMS), Pharmacists, Knowledge, Attitudes, Primary healthcare institution, Public secondary/tertiary hospital

## Abstract

**Background:**

In 2009, Chinese government launched a new healthcare reform, one of the key points of which is to establish National Essential Medicine System (NEMS). Hospital pharmacists are directly related to the implementation of NEMS. This study is to examine knowledge of and attitudes towards the implementation of the NEMS among hospital pharmacists in western China.

**Methods:**

We conducted a questionnaire survey of pharmacists from different types of medical institutions in Shaanxi Province in November 2014. We gathered demographic information about the participants, collected the data about their knowledge of and attitudes towards the implementation of NEMS, and identified the influencing factors of cognitive level. We analyzed the data and compared public secondary/tertiary hospitals and primary healthcare institutions.

**Results:**

Of the 704 participants (response rate = 70.2 %), the majority had positive and moderate knowledge (39.2 and 53.3 %) and attitudes (35.8 and 62.9 %) towards NEMS. The most participants were aware of the implementation time of NEMS (89.8 %) and zero mark-up policy (85.5 %) while the least learned of the adjustment time of National Essential Medicines List (NEML). Pharmacists from public secondary/tertiary hospitals tended to know more and have more positive attitudes. There was no statistical correlation between knowledge and attitude scores. The education level (*p* = 0.022) and number of training sessions attended (*p* = 0.028) were the only demographic variables linked to knowledge scores.

**Conclusions:**

Hospital pharmacists in Shaanxi Province had moderate knowledge of and attitudes towards the implementation of NEMS. Pharmacists from public secondary/tertiary hospitals showed better understanding. The government should therefore focus on improving the understanding of pharmacists in primary healthcare institutions and also address existing problems, especially the supply and distribution systems.

## Background

The past three decades have seen rapid economic growth in China, but no similar improvement in health services [1–3]. There has been sustainable growth in total health expenditure—5.36 % of gross domestic product (GDP) in 2012 [4]. Although the percentage of individual out-of-pocket payments is declining [5], high out-of-pocket payments still place huge financial burdens on many people [6]. To provide affordable, equitable access to high-quality health services for all citizens by 2020, the Chinese government launched a nationwide systematic reform in 2009 [7], one of the key pillars of which is the establishment and implementation of the National Essential Medicines System (NEMS). In order to make NEMS benefit the general population, this policy put great emphasis on medicines accessibility and affordability.

The NEMS is a comprehensive measure covering medicine selection, production, pricing, procurement, prescribing and reimbursement [8]. One of the most remarkable aspects of it is a zero mark-up policy for government-run primary healthcare institutions. This means that medicines included on the National Essential Medicines List (NEML) are sold to patients at cost [9]. As a result, the NEML cannot meet the specific service needs of primary health care institutions [10], such as gynecological medicines and pediatric drugs.

When all primary healthcare institutions had implemented this policy in 2012 [11], the focus shifted to county hospitals. The government has been promoting and expanding the priority use of essential medicines, as well as the proportion available in secondary and tertiary hospitals [12].

There are many literatures concerning the NEMS and a good deal of research has focused on indicators of effect, such as availability [13], affordability [14] and rational drug use [15, 16]. Some studies have also evaluated the effects of NEMS on healthcare workers [17–19], but there are still limits to the information available. For example, most research has focused on primary healthcare institutions, and there is little evidence about secondary/tertiary hospitals [18, 20]. Most studies also focus on doctors [17, 18]. Although their knowledge and attitudes are important to the implementation of NEMS, other medical staff are also closely involved.

By the end of 2014, there were 948,000 medical institutions in China, including 26,000 hospitals, 922,000 grassroots hospitals. All these medical institutions should have pharmacy departments and certain number of pharmaceutical professionals (pharmacists and pharmacy technicians), which generally accounting for 8 % of all professionals [21]. The main responsibility of hospital pharmacy is assuring the medicine quality, guiding rational drug use and making medicines be placed in proper storage conditions. The responsibility of hospital pharmacists is involving many aspects, of which the basic duty is ensuring the quality and supply of medicines and carrying out “patient-centered” clinical work [22]. Hospital pharmacists are in charge of monitoring prescriptions, dispensing medication and providing guidance on the use of medicines. They are therefore directly concerned with selection, procurement, distribution, and use of medicines [23], and play an important role in the implementation of the NEMS. In primary healthcare institutions, operating the zero mark-up policy, pharmacists actively implement the NEMS. Implementing the zero mark-up policy in public secondary and tertiary hospitals will inevitably affect the way in which pharmacists work, as well as their pay, job satisfaction and the drug supply and distribution system. After the launch of this policy, most cities established compensation policies to pay staff a basic salary plus a bonus based on performance. The performance criteria include quality of services and achieved social responsibilities such as controlling cost escalation. It is therefore very important to study the impact of NEMS on primary and higher-level hospital pharmacists.

Internationally, there are corresponding studies on pharmacists’ influences on prescribing [24], rational drug use [25] and opinions on adverse drug reaction reporting [26]. However, there are few studies on hospital pharmacists’ understanding about the NEMS. We therefore conducted a questionnaire survey of pharmacists in tertiary, secondary, and primary healthcare institutions in Shaanxi Province, western China. This aimed to examine their knowledge of and attitudes towards the implementation of the NEMS, and explore their views on problems and how these could be resolved.

## Methods

### Study design

A questionnaire was designed to probe pharmacists’ knowledge and perceptions of and attitudes towards the implementation of the NEMS in public secondary/tertiary hospitals and primary healthcare institutions. The survey was approved by the Ethical Committee of the Health Science Center, Xi’an Jiaotong University.

### Study area

The survey was conducted in Shaanxi Province [27]. Located in the western part of China, with a population of more than 37 million, it has a low to medium GDP. Shaanxi Province had a total of 181 primary healthcare institutions, 278 secondary and 48 tertiary hospitals in 2012 [4]. Shaanxi’s government implemented the NEMS in the form of a “triple unification” policy (unified bidding, unified distribution, and unified pricing) [28]. Because all primary healthcare institutions have adopted the zero mark-up policy, the Shaanxi government is now extending NEMS to secondary and tertiary hospitals.

### Sampling

The survey was undertaken in November 2014. The questionnaire was distributed at the Western Area Pharmacists Training Program, which was aiming at promoting hospital pharmacists’ professional services and rational drug use, run by the Shaanxi Health and Family Planning Commission, and attended by hospital pharmacists from across the province. Every medical institution was required to send two senior pharmacists to the training. The selected pharmacists should be familiar with the operation and management of hospital pharmacy and have at least a basic understanding of the NEMS. The organizer would afford all the charges except for the participants’ transport costs. All participants had no prior information about the survey. As a matter of convenience, we distributed the questionnaires to all attendees but only singled out public hospital pharmacists as sample. Informed consent was obtained from the training organizer. Before distributing the questionnaires, the conference host gave a brief introduction to the research team and the purpose of this survey, so that we could obtain participants’ oral agreement. Those who completed the questionnaire were considered to have consented.

### Instruments

A covering letter was attached to introduce the purpose and significance of this study. It explained that the survey was anonymous and for research purposes only, to reassure the participants of anonymity and confidentiality. The questionnaires were self-administered during break time at the conference and returned before leaving.

The questionnaire was developed specifically for hospital pharmacists and consisted of four parts. The first part asked for participants’ demographic information. The second assessed their knowledge of NEMS by six questions about the implementation time, the NEML, centralized procurement and distribution, zero mark-up policy and medical insurance reimbursement. The third part used five-point Likert scales to measure the pharmacists’ attitudes towards the implementation of NEMS. There are some barriers during the implementation of NEMS, such as the medicines in NEML are not fully meet the clinical utilization, the financial compensation of local governments is not in place or the availability of essential medicines is inadequate sometimes [28]. Therefore the final part consisted of multiple choice questions to evaluate views on the existing problems with the implementation of NEMS, and suggestions for resolving them. It included an option of “other” for new problems and solutions.

The questionnaire was pre-tested on five pharmacists at a tertiary hospital, and then adjusted slightly to ensure that it was both appropriate and easy to understand. Its validity and reliability were tested. Cronbach’s Alpha was used to validate its internal consistency and the coefficient of knowledge and attitude part were 0.81 and 0.76 respectively, giving a reasonable level of validity for 0.7 is considered as the minimum reliable level of consistency [29, 30]. Four undergraduates from the medical college were recruited and trained to carry out questionnaire distribution and data collection. The investigators had no knowledge of the research hypotheses and the correct answers to the survey questions, to avoid any potential bias in the process of data collection.

### Data entry and analysis

Completed questionnaires were encoded and the data were double-checked and verified for completeness and consistency. The data were entered into Microsoft Excel 2007 and analyzed using SPSS (version 18.0). All data were checked by two of the authors before analysis. The second and third parts were transformed into scores in Excel 2007. The knowledge part contained a mixture of right and wrong statements, with the answers classified into one of three options (yes, no and don’t know). Each correct answer was scored as 1 and a wrong or “don’t know” answer was scored as 0. The total score for all knowledge questions was calculated for each participant. For the attitude part, the points on the response scale were: 1 = “strongly disagree”, 2 = “disagree”, 3 = “neither agree nor disagree”, 4 = “agree”, and 5 = “strongly agree”. The average value was calculated for total attitude score and each statement score.

Total knowledge and attitudes scores were calculated for every participant. The possible ranges were 6 (6–0 = 6) and 24 (30–6 = 24), and these were divided into three equal parts. In order to quantify analysis, we took 60 and 80 % [31] as boundary point and also considered the balance of the score spacing. The knowledge scores were categorized as “negative” (0–2), “moderate” (3–4) and “positive” (5–6), and the attitude scores were “negative” (6–13), “moderate” (14–21) and “positive” (22–30).

The demographic information was included as public secondary/tertiary hospitals, primary healthcare institutions and overall. The knowledge and attitudes of pharmacists from public secondary/tertiary hospitals and primary healthcare institutions were compared using a chi-square test. Multiple linear regression was used to identify the determinants of knowledge scores. Bivariate correlation analysis was used to analyze the relationship between knowledge and attitude scores. A type 1 error of 0.05 was used as the threshold of statistical significance.

## Results

### Demographics

Of the 1003 questionnaires distributed, 228 from private hospital pharmacists were eliminated and 71 were unusable because of missing data, so only 704 were eligible (response rate = 70.2 %). Participants’ demographic information is summarized in Table 1. A total of 525 (74.6 %) participants were from public secondary/tertiary hospitals and 179 (25.4 %) from primary healthcare institutions; 445 (63.2 %) were female and 259 (36.8 %) were male. Similar numbers of participants had a bachelor’s degree (42.0 %, 296/704) or a college diploma (40.1 %, 282/704). Only 29 (4.1 %) had a master’s degree or above. Half of the participants (50.9 %, 358/704) had a title of intermediate technician. The amount of training received varied: none (33.2 %, 234/704), 1–2 courses (55.4 %, 390/704), 3–5 courses (9.9 %, 70/704) and more than 5 courses (1.4 %, 10/704). Education level (*p* < 0.001) and technician title (*p* < 0.001) were the only demographic variables that were significantly different in public secondary/tertiary hospitals and primary healthcare institutions (Table 1).

**Table 1:** Demographic characteristics of hospital pharmacists in Shaanxi Province

Demographic information	Public secondary/tertiary hospitals *n* (%)	Primary healthcare institutions *n* (%)	Overall *n* (%)	*p*-value
Sex				0.324
Male	199 (37.9)	60 (33.5)	259 (36.8)	
Female	326 (62.1)	119 (66.5)	445 (63.2)	
Age group (years)				0.516
≤ 25	36 (6.9)	10 (5.6)	46 (6.5)	
26–35	161 (30.7)	61 (34.1)	222 (31.5)	
36–45	186 (35.4)	67 (37.4)	253 (35.9)	
46–55	125 (23.8)	33 (18.4)	158 (22.4)	
≥ 56	17 (3.2)	8 (4.5)	25 (3.6)	
Working years group				0.272
≤ 5	101 (19.2)	28 (15.6)	129 (18.3)	
6–10	70 (13.3)	35 (19.6)	105 (14.9)	
11–20	147 (28.0)	53 (29.6)	200 (28.4)	
21–30	164 (31.2)	50 (27.9)	214 (30.4)	
≥ 31	43 (8.2)	13 (7.3)	56 (8.0)	
Education level				<0.001
Master and above	28 (5.3)	1 (0.6)	29 (4.1)	
Bachelor	251 (47.8)	45 (25.1)	296 (42.0)	
College	182 (34.7)	100 (55.9)	282 (40.1)	
Secondary school or below	64 (12.2)	33 (18.4)	97 (13.8)	
Technical title				<0.001
Senior	96 (18.3)	9 (5.0)	105 (14.9)	
Intermediate	275 (52.4)	83 (46.4)	358 (50.9)	
Junior	144 (27.4)	75 (41.9)	219 (31.1)	
None	10 (1.9)	12 (6.7)	22 (3.1)	
Number of training sessions received				0.068
None	160 (30.5)	74 (41.3)	234 (33.2)	
1–2 courses	303 (57.7)	87 (48.6)	390 (55.4)	
3–5 courses	54 (10.3)	16 (8.9)	70 (9.9)	
More than 5 courses	8 (1.5)	2 (1.1)	10 (1.4)	

All *p*-values are based on chi-square analysis

### Knowledge of hospital pharmacists about NEMS

The mean value for the total knowledge score was 4.09 ± 1.11. This was classified as “moderate”. Positive knowledge was achieved by 39.2 % of the participants, with 53.3 % achieving moderate knowledge, and 7.5 % negative. Coincidentally, 7.5 % of participants also obtained the maximum scores. The majority of participants were aware of the official implementation time of NEMS (86.8 %, 611/704), zero mark-up policy (85.5 %, 602/704), supply pattern (77.7 %, 547/704) and medical insurance reimbursement (76.8 %, 541/704). Fewest participants (21.4 %, 151/704) knew about the adjustment time of the NEML. Hence, their knowledge level was acceptable except about the adjustment time of the NEML. There was a significant difference (*p* = 0.003) in knowledge about the zero mark-up policy between public secondary/tertiary hospitals and primary healthcare institutions (Table 2).

**Table 2:** Knowledge level of hospital pharmacists about the NEMS

Items	Correct answers *n* (%)			*p*-value
	Public secondary/tertiary hospitals	Primary healthcare institutions	Overall	
Q10. The official implementation time of NEMS is 2009.	455 (86.7)	156 (87.2)	611 (86.8)	0.869
Q11. National Essential Medicines List (NEML) is adjusted every 2 years in principle.	113 (21.5)	38 (21.2)	151 (21.4)	0.934
Q12. There are a total of 307 drugs in the latest version of “National Essential Medicines List”.	319 (60.8)	107 (59.8)	426 (60.5)	0.816
Q13. Essential medicines are for provincial centralized procurement and unified distribution	415 (79.0)	132 (73.7)	547 (77.7)	0.141
Q14. Zero mark-up policy is implemented in the NEMS.	461 (87.8)	141 (78.8)	602 (85.5)	0.003
Q15. Medicare insurance reimbursement of essential medicines is the same as non-essential medicines.	404 (77.0)	137 (76.5)	541 (76.8)	0.909

### Sources of information about NEMS

The majority of the research participants reported that they had learned about NEMS through conferences (71.0 %, 500/704) followed by the Internet (42.3 %, 298/704) and journals (30.3 %, 213/704). Only 189 (26.8 %) participants obtained information from traditional media and 57 (8.1 %) obtained information from other sources, such as brochures, government documents and professional books.

### Attitudes towards the implementation of NEMS

The average value of total attitudinal score was 20.36 ± 2.96 and the range was from 11 to 30. The categories were 35.8 % positive, 62.9 % moderate and 1.3 % negative. Approximately two thirds of the sample approved (strongly agree: 8.4 %, 59/704 and agree: 55.1 %, 388/704) of improving rational drug use while less than half (strongly agree: 4.4 %, 31/704 and agree: 31.5 %, 222/704) thought that the effect of implementation of NEMS at present was satisfactory. Participants were least likely to agree that NEMS would affect staff income (strongly agree: 6.0 %, 42/704 and agree: 30.4 %, 214/704). Attitudes were not significantly different between public secondary/tertiary hospitals and primary healthcare institutions (Table 3). Bivariate correlation analysis showed there was no correlation between knowledge and attitude scores (*p* > 0.05).

**Table 3:** Attitudes towards the implementation of NEMS

Items	Public secondary/tertiary hospitals *n* (%)	Primary healthcare institutions *n* (%)	Overall *n* (%)	Mean ± SD	*p*-value
Q17. The effect of implementation of NEMS is satisfactory at present.				3.28 ± 0.76	0.419
Strongly agree	22 (4.2)	9 (5.0)	31 (4.4)		
Agree	156 (29.7)	66 (36.9)	222 (31.5)		
Neither agree nor disagree	294 (56.0)	86 (48.0)	380 (54.0)		
Disagree	41 (7.8)	14 (7.8)	55 (7.8)		
Strongly disagree	12 (2.3)	4 (2.2)	16 (2.3)		
Q18. The implementation of NEMS could guarantee the basic medical needs of the population.				3.50 ± 0.80	0.812
Strongly agree	36 (6.9)	16 (8.9)	52 (7.4)		
Agree	243 (46.3)	86 (48.0)	329 (46.7)		
Neither agree nor disagree	189 (36.0)	61 (34.1)	250 (35.5)		
Disagree	52 (9.9)	15 (8.4)	67 (9.5)		
Strongly disagree	5 (1.0)	1 (0.6)	6 (0.9)		
Q19. The implementation of NEMS could reduce the healthcare burden of the population.				3.61 ± 0.85	0.425
Strongly agree	55 (10.5)	23 (12.8)	78 (11.1)		
Agree	273 (52.0)	88 (49.2)	361 (51.3)		
Neither agree nor disagree	135 (25.7)	50 (27.9)	185 (26.3)		
Disagree	59 (11.2)	15 (8.4)	74 (10.5)		
Strongly disagree	3 (0.6)	3 (1.7)	6 (0.9)		
Q20. The implementation of NEMS could improve rational drug use.				3.63 ± 0.76	0.106
Strongly agree	39 (7.4)	20 (11.2)	59 (8.4)		
Agree	284 (54.1)	104 (58.1)	388 (55.1)		
Neither agree nor disagree	164 (31.2)	39 (21.8)	203 (28.8)		
Disagree	35 (6.7)	14 (7.8)	49 (7.0)		
Strongly disagree	3 (0.6)	2 (1.1)	5 (0.7)		
Q21. The implementation of NEMS could change the working mode of hospital pharmacy.				3.27 ± 0.86	0.065
Strongly agree	21 (4.0)	9 (5.0)	30 (4.3)		
Agree	195 (37.1)	85 (47.5)	280 (39.8)		
Neither agree nor disagree	191 (36.4)	59 (33.0)	250 (35.5)		
Disagree	111 (21.1)	25 (14.0)	136 (19.3)		
Strongly disagree	7 (1.3)	1 (0.6)	8 (1.1)		
Q22. The implementation of NEMS could reduce the medical staff’s incomes.				3.07 ± 0.97	0.653
Strongly agree	29 (5.5)	13 (7.3)	42 (6.0)		
Agree	165 (31.4)	49 (27.4)	214 (30.4)		
Neither agree nor disagree	157 (29.9)	57 (31.8)	214 (30.4)		
Disagree	159 (30.3)	57 (31.8)	216 (30.7)		
Strongly disagree	15 (2.9)	3 (1.7)	18 (2.6)		

### Effect of demographic factors on knowledge about NEMS

Table 4 shows the association between knowledge scores and demographic variables among the study population in the multiple linear regression analysis. Knowledge about NEMS was significantly associated with education level (*p* = 0.022) and the number of training sessions received (*p* = 0.028). In the complete model (Model 2), study participants’ knowledge scores were positively related to education level (β 0.131; 95 % confidence interval [CI] 0.019–0.242) and the number of training sessions received (β 0.145; 95 % CI 0.016–0.274).

**Table 4:** Multiple linear regression analysis showing predictors of knowledge levels on the NEMS

No.	β	*p*-value	95 % CI of β
Model 1			
Constant	3.806	<0.001	3.530–4.082
Education level	0.142	0.012	0.031–0.254
Model 2			
Constant	3.573	<0.001	3.229–3.917
Education level	0.131	0.022	0.019–0.242
Number of training sessions received	0.145	0.028	0.016–0.274

*CI* confidence interval

### Problems with the implementation of NEMS, and solutions to address them

A total of 591 (83.9 %) participants regarded the supply and distribution system as a big problem, followed by insufficient government funding (65.5 %, 461/704) and insufficient number of essential medicine types (60.8 %, 428/704). Only 174 (24.7 %) of participants agreed that the way in which the NEML was determined was a problem.

In relating to suggestions for improving the implementation of NEMS, most participants approved of rational selection and use (84.7 %, 596/704) and reliable systems (87.9 %, 619/704). More than half favored sustainable funding (67.2 %, 473/704) and affordable prices (58.4 %, 411/704).

## Discussion

Five years after the launch of the healthcare reforms and implementation of the NEMS, some achievements have been made [32, 33]. This study has shown that hospital pharmacists have moderate knowledge of and attitudes towards NEMS. This finding is similar to the studies [18, 19] targeting on doctors and the medical staff in primary hospital. And basically all the respondents learn of the NEMS in their studies. Xu Y et al. [20] found that medical staff had much knowledge of types and therapeutic efficacy of essential medicines. In our study, the hospital pharmacists had a good knowledge of implementation time, zero mark-up policy, supply pattern and reimbursement. However, the majority have no idea of the adjustment time of the NEML. The relevant questionnaire item was an incorrect statement that the NEML is adjusted every 2 years. This may be a result of confusion about action in China and from the World Health Organization (WHO). WHO sets a new essential medicine list at least every 2 years [34], while China operates a system of dynamic management, stipulating that NEML should be adjusted every 3 years in principle [35]. Although zero mark-up policy had in theory been applied in all primary healthcare institutions, there were still some primary pharmacists who did not know about this policy. This suggests that the NEMS had not been promoted effectively. Attitudes towards the implementation of NEMS were generally classified as “moderate”. Surprisingly, only fewer than half of the hospital pharmacists considered the effects of NEMS were satisfactory at present. Identifying the fundamental causes of the dissatisfaction would need more researches. Participants did not agree that their income was affected, but this may be because some were reluctant to provide accurate information, because this is a sensitive topic. This might have introduced a slight error.

Previous study [19] reported that the cognition level is dependent with age, education level, specialty and work experience. Inconsistently, we found that only education level and number of training sessions attended were significantly related to knowledge scores. Pharmacists in public secondary/tertiary hospitals performed better than their counterparts, this can be explained by the fact that someone with a higher education level, or who receives more training, is likely to have a better level of knowledge. Public secondary/tertiary hospitals tended to have more highly educated professionals and training opportunities than primary healthcare institutions. The government could therefore improve pharmacists’ understanding of NEMS by emphasizing the importance of talent introduction and training. More attention should also be paid to primary healthcare institutions where NEMS has been fully implemented.

Pharmacists reported some problems in the implementation of NEMS. The most frequently-reported problem was the supply and distribution system. Similar results have been found in other developing countries [36], and this seems to be a global challenge [37–39]. Problems with timely supply and distribution of essential medicines have serious consequences, such as drug shortages. As a result, some patients with mild conditions have to attend secondary or tertiary hospitals [40], which occupies scarce medical resources and increases patients’ financial burden [32]. Some participants also thought that government funding was insufficient. In fact, the government has regularly increased funding [41], but there may be a cognition gap between the government and pharmacists. Some participants added their own comments. Some questioned the principles of selecting essential medicines. They pointed out that some cheap drugs with general clinical effects were included in the NEML while a number of branded drugs with good clinical effects and high prices were not, which needs urgent improvement.

Access to medicine is the key function of NEMS. The WHO has set out a four-part framework [42] to guide and coordinate collective action on access to essential medicines. This includes rational selection and use, affordable prices, sustainable financing and reliable supply systems. The suggestions included in the questionnaire covered these four areas. The majority of hospital pharmacists suggested that reliable systems and rational selection and use were directly related to their work responsibilities. It is therefore vital to introduce evidence-based selection and pharmacoeconomics into the development of NEML. Some participants suggested other solutions, including raising doctors’ awareness of essential medicines, price management for essential medicines and standardization of the distribution system.

Although there have been some studies of hospital pharmacists, ours is the first to survey a whole province hospital pharmacists’ knowledge of and attitudes towards the NEMS in China. We took advantage of a training program targeting hospital pharmacists across the whole province to conduct the survey. This unique opportunity to select participants has greatly improved the representativeness of the survey; meanwhile, the sampling method may cause a selection bias. This study also has some other limitations, and its results should be interpreted with caution. The survey was conducted only in Shaanxi Province, which may not be typical of the wider situation in China. Nevertheless, we chose Shaanxi Province as sample district because it is broadly representative of the typical health and health system status of the 12 western provinces of China [27], and the total provincial GDP was US$288 billion in 2014, ranking in the middle level in China. It was conducted in a training meeting, so a few participants probably completed the questionnaire in consultation with others. Such behavior would bias the results somewhat. However, our study still provides crucial evidence on hospital pharmacists’ knowledge of and attitudes towards the implementation of NEMS, and the differences between pharmacists in public secondary/tertiary hospitals and in primary healthcare institutions.

## Conclusions

Overall, hospital pharmacists in Shaanxi Province showed moderate knowledge and attitude levels about the implementation of NEMS. Compared with those in primary healthcare institutions, pharmacists in public secondary/tertiary hospitals performed better, probably because they were more likely to have a higher level of education and receive more training. There is therefore an urgent need for governments to provide increased opportunities for further education and training for medical institutions, especially primary healthcare institutions. Some problems still exist with the implementation of NEMS, and particularly with the supply and distribution system. Overall, hospital pharmacists deemed reliable systems to be the most important issue to improve the NEMS.

## Abbreviations

GDP, gross domestic product; NEML, National Essential Medicines List; NEMS, National Essential Medicine System; WHO, World Health Organization
